# The Doctor in Fiction

**Published:** 1910-05-07

**Authors:** 


### The Practitioner's Relaxations.

### THE DOCTOR IN FICTION.

If the characters of fiction were to be classified according to profession or business it would probably be found that the medical and surgical class has received a greater variety of treatment than can be claimed by any other section. To account for this it is only necessary to remember the important part played by the medical man in the average human life-story. As president over life and death he is ubiquitous in fiction as in fact, and the diversity of his literary portraits is perhaps only a natural result of the frequency of his occurrence in association with those incidents of life which are the raw material of the literary artist. By reason of this diversity it is impossible to find a basis for any sweeping generalisations on our subject, and the most that can be done is to indicate a few well-defined types.

The observance of professional etiquette tends to product a spirit of conservatism, whilst frequent observation of the " open sores of society " leads to sympathy with revolutionary ideas. Accentuation of these tendencies has produced two large groups of the medical profession's representatives in fiction. These two groups are open to some sub-division: there are conservatives of the mellow type and others whose conservatism has no more substantial basis than lack of experience and imagination.

The revolutionary type occurs most frequently in modern literature. There is an interesting example in Gorky's " Comrades," Ivan Danilovitch by name. He is described as having a high-pitched, thin, nervous voice, winking eyes, unruly hair, and a habit of twisting his beard. In striking contrast we have the conservative doctor in Tolstoy's " Fruits of Culture," described as healthy, fat, red-faced, and loud-voiced, with a self-satisfied smile continually on his lips. Before passing from Russian fiction there is a unique example to be noted in Gogol's comedy, " The Inspector-General." Here we are shown a hospital physician who does not know a word of his patient's language, and whose sole utterance throughout the play is a single grunt. Some other amusing details are given of the primitive state of affairs in this truly Russian institution.

The novels of Zola have lost much of their original vogue, probably because their author was too much of a propagandist to preserve the lightness of touch and impartiality of judgment which are necessary for the production of fine art. Nevertheless, he will always receive a certain amount of attention because of his merits as a literary photographer. In his " Fécondite " we are introduced to two interesting and highly contrasted physicians, Drs. Boutan and Gaude. The former is portrayed as a family practitioner of the conservative type, whilst Dr. Gaude is shown as a rash experimenter, whose patients, after deriving great temporary benefit from his operations and so gaining him a fashionable reputation, gradually fall into a state of collapse. Zola's Doctor Pascal and the medical student in " La Joie de Vivre " are also well worthy of note. The country physician who was the husband of Flaubert's Madame Bovary is an interesting study of a stolid and unambitious type. Urged on to make an enterprising bid for fame, he merely meets with failure.

Honoré de Balzac, perhaps the greatest of French novelists, frequently introduced medical characters into his work. In his " Messe d'Athée " there is a fine picture of a successful Parisian specialist, whose harsh and ungentle exterior concealed a vein of tenderness, which was the result of the touching fidelity of a poor water-carrier who had helped him in his struggling student days.

One of the earliest English representatives of the profession in fiction was drawn by the pen of a man whose outlook on humanity had a similar breadth to that of the author of the " Comédie Humaine." In the " Canterbury Tales " is the well-known description of a " doctour of phisyk " who was among the pilgrims.

In modern English fiction two books of this class may be singled out for mention. Thomas Hardy's " The Woodlanders " should be read for its portrait of Dr. Fitzpiers, a very typical modern. Sir A. Conan Doyle's " Round the Red Lamp " is a good book of medical short stories, one of which is a literary version of the theme used by the Hon. John Collier in his picture " Sentence of Death," which created so much sensation a few years ago. It contains also some good portraiture of the medical student.

Residents in the Manchester district will need no recommendation to Dr. Sackville Martin's amusing hospital comedy, " Cupid and the Styx," which has been frequently played there by Miss Horniman's repertory company. It deserves to be more widely known. The plot is concerned with the attraction of two house surgeons by a hospital nurse, who eventually marries the consulting physician. The strength of the play lies in its delineation of character and its sidelights on hospital life.

